# A Narrative Review on Prevention and Early Intervention of Challenging Behaviors in Children with a Special Emphasis on COVID-19 Times

**DOI:** 10.2147/PRBM.S354428

**Published:** 2022-06-22

**Authors:** Sarah Musa, Ismail Dergaa

**Affiliations:** 1 Department of Preventative Health, Primary Health Care Corporation, Doha, Qatar

**Keywords:** COVID-19, psychosocial wellbeing mental health, Pyramid Model, family-centered intervention, children, adolescents

## Abstract

**Background:**

COVID-19 and the measures stemming from efforts to control it have affected the psychosocial wellbeing of children and adolescents. The increasing trend of challenging behavior has exerted further pressure on parents and schools. Understanding socioemotional development and interrelating triggers is the key to management. Early interventions prevent the future threat of mental illness and risky acts. Effective strategies are ones that primarily focus on strengthening parent–child interactions.

**Aim:**

The purpose of this paper is to review the literature on the (i) psychosocial and behavioral impacts of COVID-19 on children/adolescents and (ii) approaches to identify determinants of challenging behaviors as a principal guide to effective interventional strategies for children and their families.

**Methodology:**

Electronic database searches of PubMed, ScienceDirect, Medline, and Scopus were conducted to identify studies meeting the inclusion criteria that address the impact of COVID-19 on behaviors, contributing factors, and management in the context of families/schools. The content of the selected articles was themed under five categories, namely the developmental milestones, the Pyramid Model, the Positive Behavioral Support, the management strategies, and the impact of COVID-19 on children/adolescents’ behavior.

**Results:**

The present review demonstrates considerable influence of COVID-19 on children and adolescents’ behavior and mental wellbeing. It stresses the importance of early family-based interventions focusing on the triggers of challenging behavior. Functional Behavioral Assessment and Behavioral Intervention Plan provide a systematic analysis with a strategic plan that support children’s self-regulation and socioemotional intelligence. Regular behavioral screening is vital to promote prevention and early management.

**Conclusion:**

Managing behavioral difficulties remains an area of deficit for parents, teachers, and health care providers. With a quality support, parents and schools will be able to clearly characterize the challenging behavior, understand the causes, reinforce parent–child interactions, and consequently, gain the strategic skills required to apply it within natural settings. Timely interventions will limit the risk of future misconduct and mental disorders.

## Background

### Definitions and Concepts

Behavior can be described as challenging when it exhibits a recurrent pattern or interferes with or is at risk of interfering with optimal learning or engagement in prosocial interactions with peers and adults.^1^ Emotional dysregulation and challenging behaviors may adversely impact interpersonal competences and academic performance, leaving the child with enduring effects.^2^ However, cultural sensitivity led to variations in distinguishing what is deemed inappropriate behavior considering the different norms and beliefs.^3^,^4^

### Categories of Challenging Behaviors

Behavioral problems have been grouped into two broad spectrums: internalizing and externalizing.^5^,^6^ Internalizing behaviors are expressed inward and often go undetected, such as difficulty concentrating, being anxious, persistent avoidance of activities, social withdrawal, crying or hiding.^6^,^7^ Externalizing behaviors are expressed toward the outer environment such as hitting, spitting, property destruction, fleeing and yelling.^6^,^7^

However, it is not uncommon for children and adolescents to exhibit co-occurring disorders of both types.^8^ For instance, food insecurity, homelessness, exposure to violence, abuse or neglect may lead to both internalizing (eg, being secretive, self-conscious, experiencing aches and pain, anxious, fearful) and externalizing challenging behaviors (eg, exaggerated startle responses, aggression, bullying or fighting).^6–9^

### The Impact of COVID-19 on Behaviors

COVID-19 and the imposed restrictive measures have affected to a large-scale the psychological wellbeing of children and adolescents.^10^ Disruption of daily routines and shifting into remote learning have resulted in an increased frequency or severity of challenging behaviors, representing a particular source of stress to parents/families.^11^ A study conducted during the early stages of the pandemic in the Philippines indicated that having a higher number of children in the family was positively associated with a higher level of psychological distress.^12^ Furthermore, Ren et al^13^ in their study evaluated the psychological impact of COVID-19 following school reopening and found that 32.4% of students showed symptoms of depression, while 15.5% exhibited anxiety symptoms. The study also revealed that those at higher grades and fears of being infected were at greater risk of adverse psychological outcomes.^13^

Comparative results were obtained even at later stages of the pandemic. Among 1771 adolescents in China, depression and anxiety were estimated to be 30.8% and 28.3%, respectively. Sleep quality, resilience, social/school status, perceived social support, and adaptive coping strategies were amongst the protective factors, while maladaptive coping strategies were a risk factor.^14^

Challenging behaviors are relatively prevalent, even prior to the emergence of COVID-19. A study in Boulder County, USA, indicated that yells and screams (73%), hurting oneself/others (69.5%), and getting irritable/frustrated easily (66.1%) were amongst the most common challenging behaviors reported by parents.^15^ In New Zealand, a parental survey of 10,457 children aged 3 to 14 years revealed that 8% of children had substantial social, emotional, or behavioral issues, while 7% had a “borderline” score.^16^

### Determinants of Behaviors

Several interrelated factors have been shown to influence children’s behavior, including developmental, environmental, and socio-cultural aspects.^17^ The prevalence of challenging behaviors in young children was estimated to be about 10% and may reach as high as 25% for those coming from low-income families.^17^,^18^

Preschoolers are three times more likely to be expelled from a childcare program due to active behavioral problems, as compared to grades K12.^19^ Aggressive and antisocial behaviors may persist beyond the age of three in about 3 to 15% of preschool-age children.^20^ Approximately, half of these children are embarking on a path that will inevitably lead to delinquency and criminal acts in adolescence and adulthood.^21^

### Consequences of Challenging Behaviors

While some children may outgrow this kind of behavior by the time of school entry, others demonstrate persistent and even intensifying patterns, leading to academic failure and social maladjustment.^22^ Fifty percent or more of toddlers and preschoolers with disruptive disorders were found to exhibit challenging behaviors at least up to four years later.^16^

In a qualitative study conducted by Fox et al^17^ families reported that behavioral problems invariably impacted the family structure, routines, and activities. The conclusion of the study has given support to the system perspective, which considers children and family struggles to be the product of interconnected family situations rather than a single environmental element.^23^

Disruption of daily routines, home confinement, lack of coping strategies, and changes in sleeping and eating patterns over the period of the COVID-19 pandemic are likely associated with rising rates of mental health challenges, most markedly among children, adolescents, and their families.^24^ Evidence from several studies^25–30^ indicated the importance of early management of behavioral problems in prevention of future risks and adverse outcomes. For example, mental health difficulties, physical health burden, relationship and parenting problems, substance abuse and sexual risk taking.^25–30^ Hence, structured regulation strategies tackling emotional, behavioral, and psychological perspectives are pivotal to mitigate the adverse effects of the pandemic. Understanding the factors that influence behavior is useful for successfully implementing effective interventions.

Thus, the present review aims to synthesize the available literature on (i) the impact of COVID-19 on children and adolescents’ behaviors (ii) determinants of challenging behaviors in relation to environment and social-emotional development; and (iii) a family-centered strategic interventional framework for the management of such behaviors.

## Methodology

### Search Strategy

We reviewed the literature pertaining to determinants and intervention strategies aimed at managing challenging behaviors among children and adolescents. We conducted an electronic search for studies from July 2021 through September 2021, using the following electronic databases: PubMed, ScienceDirect, Medline, and Scopus. A combination of the following keywords was used to search for titles and abstracts: “challenging behavior” OR “maladaptive behavior” OR “social-emotional” OR “internalizing” OR “externalizing” OR “children” OR “adolescents” OR “Pyramid Model” OR “COVID-19” OR “Behavioral intervention” OR “positive behavioral support” OR “family-centered” OR “school”. We then conducted hand searches using reference sections from retrieved articles. To maximize the potential of studies included no restriction to publication date or language was applied.

### Selection Process

The inclusion criteria established for the selection of the articles were: (1) focusing on children and adolescents’ behavior up to 18 years old; (2) addressing developmental and socio-emotional determinants of challenging behavior; (3) discussing the impact of COVID-19 on children/adolescents’ behavior; (4) Provide assessment of challenging behavior using Positive Behavioral Support, and (5) manage in the context of families and/or schools. Articles were excluded from the review if they examined children with developmental or mental disabilities due to the likelihood of interference with the prevalence, severity, assessment, or management of challenging behaviors.

### Data Collection Process

Two independent reviewers were involved in the database search, and any disagreement was resolved by discussion or by a third reviewer. Articles that met the selection criteria were retrieved for this review and the relevant contents of the articles were divided into five theoretical categories, including: (i) developmental milestones; (ii) the Pyramid Model; (iii) Positive Behavioral Support; (iv) management strategies for challenging behavior in the context of families and schools; and (v) the impact of COVID-19 on children’s or adolescents’ behavior.

## Results

### Behaviors Relative to Developmental Milestones

It is critical to recognize the age-appropriate childhood developmental milestones (ie, motor, verbal, social, emotional, and cognitive skills) as markers for behavioral acts, particularly throughout the transition into more advanced milestones.^31^ Some challenging behaviors are developmentally appropriate for youngsters as they gain new abilities and progress through life stages.^32^ For example, the peak of physical aggression between the ages of 17 and 42 months is considered typical in this developmental period.^33^

Transitions such as separation from parents, attempting to be more independent, or frustration due to a lack of abilities may stimulate emotional distress.^34^ Nevertheless, it is important to acknowledge that every behavior serves a function or purpose. For example, communication difficulties such as delayed language or speech or poor social competence may trigger challenging behavior as a way of communicating with their environment, especially during anxious or stressful situations.^35^

### The Pyramid Model

**Figure 1:**
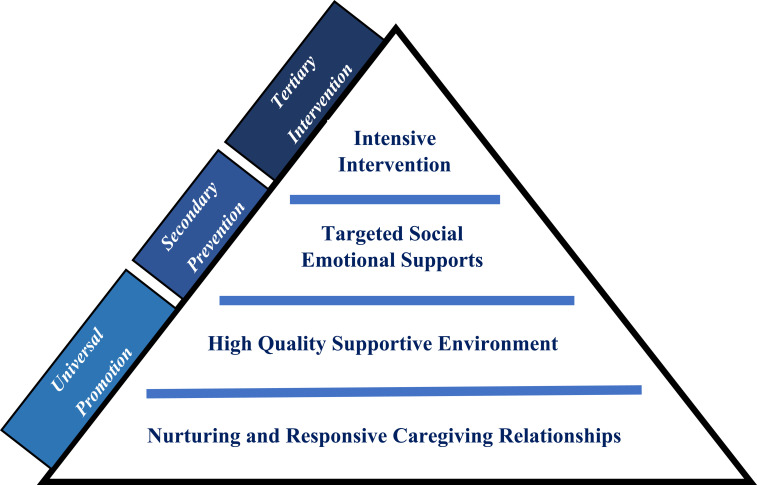
The Pyramid Model.

The Pyramid Model is an evidence-based, multi-tiered framework that supports young children’s social, emotional, and behavioral development in early years settings.^36^ It is conceptualized to provide three levels of intervention practice: universal promotion for all children, secondary preventions for those at risk of social emotional delays, and tertiary interventions for those with persisting behavioral problems^37^ (Figure 1).

#### Tier 1: Universal Promotion

- Nurturing and responsive relationships: Enhancing school-family partnerships and setting mutual goals is a key to effective solutions.^38^,^39^ The Centers for Disease Control and Prevention (CDC) considered nurturing relationship as a primary component of early childhood education that promote positive health-related outcomes especially for at-risk children.^39^
- High quality supportive environments: Appropriate behaviors are best aroused within a stimulating, consistent and interactive learning environment.^40^ When kids are taught rules and expectations, challenging behaviors are replaced with opportunities. In an attempt to correct a particular behavior, excellent practice advises that five or more efforts to praise children for positive behavior are needed.^41^

#### Tier 2: Secondary Prevention

Targeted social-emotional support: the focus of this level is to support social skills and emotional regulation, especially for at risk children who need more systematic and focused instructions.^41^ Children are assisted in expressing their emotions, improving problem solving skills, cooperative responding, peer interaction, and dealing with negative emotions such as anger.^41–43^ For example, parents and teachers can lead activities through behavioral modeling and role-play with positive reinforcement strategies when a desired behavior is demonstrated.^41^

#### Tier 3: Tertiary Interventions

Individualized intensive intervention: children with persistent behavioral problems not responding to previous tiers are offered a rigorous, tailored intervention using PBS. Progress is continuously monitored in relation to specific pre-determined goals.^44^,^45^

### Positive Behavioral Support (PBS)

PBS is a person-centered, evidence-based strategy to assist children with behavioral problems in a variety of settings.^46^ It deems challenging behavior as a product of multiple interactive variables of interpersonal relationships, physical environment, reactions of others and the way support is provided.^47^ Individual factors such as trauma, intellectual disability, general health, and mental health should all be considered.^48^,^49^

PBS is most effective when planned strategies are implemented in a consistent manner.^50^ Families and teachers are encouraged to work together to achieve the level of fidelity required to produce desirable outcomes.^50^,^51^ PBS consists of four main steps: FBA; developing a hypothesis about why the behavior is happening; undertaking a functional behavior analysis to test the hypothesis; and developing a BIP.^51^,^52^

### Functional Behavioral Assessment (FBA)

FBA is a method used to identify the associations between physiological or environmental factors and behavioral problems.^53^ The goal is to detect variables related to the occurrence of a specific behavior and to determine the function or purpose of that behavior in relation to one of four categories:^53^ social attention, escape, tangible, ie, the desire for certain things, or sensory, ie, internally rewarding or assisting in coping with negative emotions such as boredom or anxiety.

Topography, incidence, and duration of behavioral problems are identified through interviews, observations, and analysis.^54^ Precedent events that occur prior to the problematic behaviors are outlined, as are the consequences that maintain the behavior.^54^ Successful implementation of PBS was shown to be effective in minimizing challenging behavior through enhancement of new target skills.^55–59^

Behavior is assessed broadly in three stages: indirect, direct, and hypothesis testing.^60^ Indirect evaluation includes gathering information from existing databases through interviews with parents, teachers, or peers.^60^ The key is to establish a valid definition of the target behavior. For example, a defiant tantrum can be expressed by throwing materials off the desk, folding the arm and/or using inappropriate language. In the direct stage, extended analysis is carried out to identify frequency, duration, topography, and the environment in which the behavior occurs. In the final stage, hypothesis testing aims to translate the findings of previous steps into an A-B-C statement that addresses the causes of the problematic behavior, the consequences that reinforce a behavior, and the provision of replacement behaviors. Examples of ABC observation for challenging behavior are illustrated in (Table 1) and real-world scenario examples of ABC behavior are shown in (Table 2).

**Table 1:** Examples of ABC Observation for Challenging Behavior

Antecedent	Behavior	Consequences
Unstructured social situations (eg, free time, sports, games, family events)	Repetitive comments or questions, crying, dropping to the floor, hitting others	Avoids situations and social demands
Difficulty completing tasks with precision (eg, makes errors)	Crying, throwing, or destroying materials, refusing to complete activities	Obtains assistance or task is modified or delayed
Asked to do multiplication and long division problems, sitting next to Ahmed	Talking in class, arguing with Mr. Aaron (math teacher)	Gets sent to the principal’s (Mr. Michael’s) office

**Table 2:** Real Scenario Examples of ABC Observation

Antecedent	Behavior	Consequences	Function/Purpose
Noora does not enjoy/like group activities	Noora hits the person in the group sitting nearest to her	The teacher takes Noora to sit outside in the quiet corridor. Noora learns that when she wants to be taken out of a group activity, she hits someone and gets removed from the class	Escape
Father attempt to leave the house/go to work	Sarah screams every instance that her father walks away from her.	Her father returns to her and asks, “What’s wrong Sarah. Sarah learns whenever she wants her father to stay with her, she screams.	Attention
Khalid’s preferred toy is played by his brother	Become aggressive, hits his brother and scream	Mother gives Khalid back his toy and told his brother do not use this toy again. Khalid learns to not share his toys and hit his brother whenever he uses his toys.	Tangible
Maha is sitting in class, the teacher is not paying attention to her nor given anything to do	Maha starts rocking, scripting, and hand-flapping at her disk. This movement feels good to her.	Next time Maha does not have anything to do, she will do these movements	Sensory

### Behavioral Intervention Plan (BIP)

BIP consists of multicomponent interventions that are aligned with patterns observed throughout the assessment.^61^ Given that behaviors can be context-dependent (eg, a child only hits when sibling takes away his toys) and multi-functional (eg, screaming occurs both to obtain parental attention and delay certain tasks), combined interventions are usually recommended.^61^

BIP include a clear explanation of the behavior, the relationship between cause and effect, interventions used and their outcomes, behavioral goals, a plan for supporting new behavior, a description of success, evaluation, and monitoring process.^62^,^63^

When an intervention strategy is selected, guidance might aim for either changing the antecedents and/or the consequences related to a behavior (ie, using antecedent and consequence strategies) or developing more socially appropriate and adaptive replacement skills.^63^ Focusing on behavioral triggers, antecedent interventions promote behavioral change through either eliminating or adding antecedents that ultimately reduce the likelihood of challenging behavior.^64^ Examples of ABC strategies by behavioral function are presented in (Table 3).

**Table 3:** Examples of ABC Strategies by Behavioral Function

Function	Antecedent Strategies	Replacement Skills	Consequence Strategy
Avoid non-preferred activity eg, bath time	Create visuals for evening routine with a preferred activity eg, story time at the end of the routine. Provide a choice of taking a bath, or a shower, bubbles, or no bubbles.	Teach how to request a shower instead of a bath	Provide frequent verbal praise for appropriate behavior. Celebrate the completion of a bath activity.
Escape tooth brushing	Use visual (timer) or auditory cues eg, preferred song signals to show how long the activity will take. Use a favorite flavored toothpaste	Teach how to request a break from the current activity eg, saying stop, holding up his hand	Provide frequent verbal praise for appropriate behavior or appropriate requests for short breaks. Follow appropriate tooth brushing with favorite activity.
Attention seeking eg, in class	Place student at a desk where they are easily accessible Give students intermittent attention for positive or neutral behavior	Teach students more appropriate way to ask for attention eg, as raise hand, wait patiently to call on you.	Respond quickly if a student asks appropriately. Give frequent attention for positive behavior. Appraise/award star of the week for persistent positive behavior

**Figure 2:**
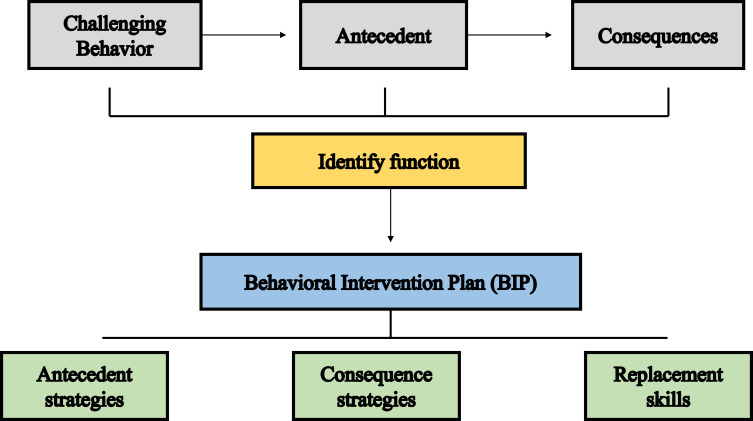
Flowchart of Functional Behavioral Assessment (FBA) and Behavioral Intervention Plan (BIP).

In school settings, interventions may include environmental modifications such as allowing students to sit in a specific location in the class or providing a quiet, distraction-free environment. Working in small groups, changing tasks, oral tests, curriculum material adaptations and group/individual counselling, are further examples.^59^,^65^ Flowchart of FBA and BIP is illustrated in (Figure 2).

### Strategies for Managing Challenging Behavior in the Context of the Family

### Responding to Challenging Behaviors

Aside from educating a child on the appropriate methods of communication, the way parents/teachers react to a particular behavior remarkably contributes to the duration, frequency, or intensity of such behavior.^58^ A message should be conveyed to the child that challenging behavior will not be successful.

The verbal or physical redirection method is a simple but highly effective strategy for shifting a child’s behavior into a more desirable one.^66^ For example, once Sarah appears ready to toss a toy when she is not getting attention, her mom can redirect her by saying, “As soon as you put away the toys, we can read your favorite bedtime story.”

### Family-Centered Intervention

Being primary caregivers, parents are considered the most valuable resources for the management of challenging behaviors.^67^ Interventions are more likely to have an impact when parental focus is shifted from consequences to reasons of misbehavior.^33^ Parental coaching aims to support parents in employing new skills when challenging behaviors occur. It is based on problem-solving skills gained through scaffolding, with an emphasis on three areas of support: cognitive, emotional, and autonomy.^57^

Family-centered methodology works on improving parents’ capacity to principally understand their child’s social and emotional cues and consequently, promote self-regulatory behavior and emotional intelligence.^68^ Natural environments such as home and school are considered the ideal settings for interventions, allowing observation of multiple interrelated factors that can affect children’s behavior.^69^,^70^ Lucyshyn et al^71^ demonstrated the significance of interventional modeling and parental coaching through problem-solving discussions, behavioral rehearsal, self-monitoring, and evaluation.

### Parental Model

The aim of the model is to foster family involvement in supporting their child’s early development. Instead of addressing behavioral problems with either parents or children independently, strategies are viewed in terms of parent-child interactions.^72^ Roggman et al^73^ proposed that parents need to be actively supported in recognizing their own resources, strengths, and needs, focusing on their own children rather than a standardized curriculum. Helping parents discuss their ideas, actions in place, and feedback with the provision of problem-solving scenarios are crucial for successful outcomes.^74^

### Parental Scaffolding

The way in which adults provide children assistance to obtain new skills as they grow through the stages of development is described as scaffolding.^75^ For instance, caregivers may breakdown a certain task into smaller, simple steps and provide elements of basic understanding that will help in the solution and actual demonstration of the task. To enhance the effectiveness of scaffolding and reduce the level of frustration caused by lack of skill, modelling, provision of hints or cues, and adapting materials may be used.^75^

Scaffolding has been identified as a high-quality parenting approach leading to favorable behavioral tendencies and self-regulation using children’s own abilities.^76^ Prior to the occurring of a challenging behavior, it is important for parents to teach their children problem solving skills instead of long speeches that the child may or may not comprehend.^77^ Three types of scaffolding have been identified, including cognitive, emotional, and autonomous.^33^

- Cognitive support.

This type of support aims to empower children to understand and apply new strategies, review problem solving steps, and realize rational underlying decisions in the direction of self-guided learning.^26^ Through effective feedback, cognitive support helps children accept different viewpoints and create a balance between pride in their abilities and recognizing the importance of reliable guidance that promotes self-confidence.^78^

- Emotional support.

Scaffolding with emotional support implies the use of positive reinforcement, verbal, and nonverbal communication to enhance emotional regulation.^33^ Mothers who help their children develop emotional literacy and teach coping strategies through play, storytelling, role modelling, taking turn, and sharing, tend to have children who are engaged in more prosocial behavior, while aggressive behaviors are associated with those who are inattentive of their child’s emotional triggers.^79^

- Support for Autonomy.

Refers as the ability of parents to provide support for their children, while preserving their independency and decision-making skills.^33^ Autonomy-promoting questions give opportunities for children to reflect on their own mental processes.^78^ For example, asking the child “How do you think we should handle this?” or “How do you feel?” enables self-expression and improve one’s sense of control. Therefore, instead of asking the child to say sorry, he/she can describe their feelings and how they believe it can be-improved. Autonomy support via stimulating parent-child interaction and minimizing judgment/control will improve problem-solving abilities, empathy, compassion, and prosocial behaviors.^33^

### Impact of Covid-19 on Children Behavior

The unexpected disruption of the social fabric and norms has affected the behavioral and mental health of the public, including children.^80–90^ The psychosocial wellbeing of children has been affected in several ways, as this unprecedented situation changed the way they typically grow, learn, play, behave, interact, and regulate emotions.

Schools’ closures, transition into remote learning, and the absence of face-to-face peer interactions have impacted important perspectives in children’s lives. Children, especially younger ones, were deprived of opportunities such as physical activity, playing and group activities, resulting in substantial disruption to critical developmental milestones.^91^

In China, Wang et al^92^ explored the psychosocial and behavioral problems of 11,072 children and adolescents in the early stages of reopening schools. Among psychosocial behaviors, parent-offspring conflict, prolonged homework time, increased sedentary behavior and screen time, sleep problems, and physical inactivity were most frequently identified. Higher internalizing and externalizing behaviors were noted, specifically, children aged 6–11 who returned to school showed more depression, compulsive behavior, and hyperactivity, while adolescents of age 12–16 showed more aggressive behavior, compared to those who were home schooled.^92^,^93^

Previous studies have demonstrated that in addition to the increase in clinging, inattentive and irritability documented at the beginning of the epidemic, with its link to disrupted school and daily routine, poor dietary habits leading to obesity, and increased use of electronic devices, can further aggravate adverse effects on children and adolescents.^94–96^

School reopening has brought a ray of hope around the world in terms of restoring the sense of structure and stimulation necessary for children’s psychosocial wellbeing.^97^,^98^ However, the readjustment period is expected to deal with several negative sequelae emerging from academic pressure, students’ relationships with teachers and peers, and difficulty adjusting to school routine.

Previous studies indicated that academic pressure driven by parents’ or teachers’ expectations, irrespective of age and sex, was amongst the most identified stressors in students.^99^,^100^

Children who were disproportionally affected including those with preexisting mental health concerns, developmental disorders, learning disabilities or any other challenges may experience greater adjustment issues and require individualized learning plan with additional support.^101^

## Limitations

This review had certain limitations, mainly attributed to the subjective nature of the narrative style literature. First, there is the possibility of misinterpretation of results and drawing conclusions (which is usually due to selection bias, subjective weighing of included studies, and unspecified data synthesis). To mitigate this limitation, we adopted several methods (ie, forming a search strategy, the process of selection and data synthesis) more characteristic of systematic reviews. Use of these methods helped to reduce selection bias by ensuring our source selection decisions were procedurally structured and precise.

The second limitation is the inclusion of a small number of studies that were conducted during the COVID-19 pandemic, creating a perception of theoretical rather than practical relevance. To overcome this limitation, we have enriched the introduction with additional post-pandemic (at early and later stage) study results to show the significance of the increasing trends and potential related mental health illness.

## Conclusion

The results of the present review highlighted the considerable impact of the COVID-19 pandemic on children/adolescents’ behaviors and their mental wellbeing. Identifying behaviors’ determinants in the context of developmental, environmental, and sociocultural remains the key step to mitigating the adverse effects such as social maladjustment, academic failure, and future risky acts. Scalable and family-based mental health interventions built on the Pyramid Model, FBA, and BIP will promote effective and sustainable outcomes.

The present review supports that enhancing parental capacity through training, coaching, and empowering them to identify their own resources and strengths will help construct positive parent-child interactions. Additional attention should be given to the children/adolescents who are more susceptible to mental health challenges through a collaborative approach involving parents, schools, healthcare providers, and mental health services.

Owing to the COVID-19 containment measures and the impediment to traditional face-to-face services, other innovative psychological supports, such as Internet Cognitive Behavioral Therapy, may be an effective alternative to reduce barriers to access mental health resources.

### Implications for Practice, Policy, and Future Research

The empirical literature synthesis focuses on several key stakeholders (parents, schools, practitioners, community, policymakers, and researchers) who work with children or adolescents. The framework of Functional Behavioral Assessment and Behavioral Intervention Plan offers a strategic template that facilitates and supports a comprehensive array of evidence-based services components, from developmental surveillance, promotion, and prevention to intensive intervention, through an individualized plan tailored to the child and family’s needs.

Professional development for practitioners, schools, and parents is important to ensure they have the adequate skills and knowledge to conduct behavioral assessments, identify determinants, socio-emotional competences, and implement effective interventions within natural environments accordingly. The current review provides an expanded understanding of the role of mental health support within the school environment, allowing for continuous monitoring and evaluation.

The results from this review have provided sufficient grounds for further longitudinal research to examine the impact of supportive environments, parental coaching, and interventions on children’s behaviors as well as the consequences of persistent challenging behavior in developing future mental health illnesses or risky acts. Another avenue for research would be to examine the adverse effects related to COVID-19 restrictive measures on the mental health of children, adolescents, and families.

## Data Sharing Statement

Not applicable because of review type of the article. All data and material are available under references.

## References

[1] Ulber J, Tomasello M. Young children’s prosocial responses toward peers and adults in two social contexts. *J Exp Child Psychol*. 2020;198:104888. doi:10.1016/j.jecp.2020.104888 32622070

[2] Shonkoff JP, Garner AS, Siegel BS, Dobbins MI, Earls MF, McGuinn L. Committee on early childhood, adoption, and dependent care. The lifelong effects of early childhood adversity and toxic stress. *Pediatrics*. 2012;129(1):e232–e246. doi:10.1542/peds.2011-2663 22201156

[3] Hornby G, Lafaele R. Barriers to parental involvement in education: an explanatory model. *Edu Rev*. 2011;63(1):37–52. doi:10.1080/00131911.2010.488049

[4] Musa S, Dergaa I, Mansy O. The puzzle of Autism in the time of COVID 19 pandemic:“Light it up Blue”. *Psychol Educ J*. 2021;58(5):1861–1873.

[5] Hinshaw SP, Han SS, Erhardt D, Huber A. Internalizing and externalizing behavior problems in preschool children: correspondence among parent and teacher ratings and behavior observations. *J Clin Child Psychol*. 1992;21(2):143–150. doi:10.1207/s15374424jccp2102_6

[6] Ter Bogt TF, van Dorsselaer SA, Monshouwer K, Verdurmen JE, Engels RC, Vollebergh WA. Body mass index and body weight perception as risk factors for internalizing and externalizing problem behavior among adolescents. *J Adolesc Health*. 2006;39(1):27–34. doi:10.1016/j.jadohealth.2005.09.007 16781958

[7] Kerig PK, Becker SP, Egan S. From internalizing to externalizing: theoretical models of the processes linking PTSD to juvenile delinquency. *PTSD*. 2010;33:78.

[8] Edwards RC, Hans SL. Infant risk factors associated with internalizing, externalizing, and co-occurring behavior problems in young children. *Dev Psychol*. 2015;51(4):489–499. doi:10.1037/a0038800 25664829

[9] Frye SS, Perfect MM, Graham JW. Internalizing disorders. In: *Handbook of Pediatric Behavioral Healthcare*. Cham: Springer; 2018:155–169.

[10] Musa S, Elyamani R, Dergaa I. COVID-19 and screen-based sedentary behaviour: systematic review of digital screen time and metabolic syndrome in adolescents. *PLoS One*. 2022;17(3):e0265560. doi:10.1371/journal.pone.0265560 35312701 PMC8936454

[11] Christner N, Essler S, Hazzam A, Paulus M. Children’s psychological well-being and problem behavior during the COVID-19 pandemic: an online study during the lockdown period in Germany. *PLoS One*. 2021;16(6):e0253473. doi:10.1371/journal.pone.0253473 34161376 PMC8221463

[12] Tee ML, Tee CA, Anlacan JP, et al. Psychological impact of COVID-19 pandemic in the Philippines. *J Affect Disord*. 2020;277:379–391. doi:10.1016/j.jad.2020.08.043 32861839 PMC7444468

[13] Ren Z, Xin Y, Ge J, et al. Psychological impact of COVID-19 on college students after school reopening: a cross-sectional study based on machine learning. *Front Psychol*. 2021;12:641806. doi:10.3389/fpsyg.2021.641806 33995195 PMC8116564

[14] Ren Z, Xin Y, Wang Z, Liu D, Ho RCM, Ho CSH. What factors are most closely associated with mood disorders in adolescents during the COVID-19 pandemic? A cross-sectional study based on 1771 adolescents in Shandong Province, China. *Front Psychiatry*. 2021;12:728278. doi:10.3389/fpsyt.2021.728278 34603106 PMC8481827

[15] Trigg AB, Keyes AW. Child care and early education as contexts for infant mental health. In: *Handbook of Infant Mental Health*; 2019:581–590.

[16] D’Souza S, Underwood L, Peterson ER, Morton S, Waldie KE. Persistence and change in behavioural problems during early childhood. *BMC Pediatr*. 2019;19(1):259. doi:10.1186/s12887-019-1631-3 31349812 PMC6659228

[17] Ogundele MO. Behavioural and emotional disorders in childhood: a brief overview for paediatricians. *World J Clin Pediatr*. 2018;7(1):9–26. doi:10.5409/wjcp.v7.i1.9 29456928 PMC5803568

[18] Webster-Stratton C, Hammond M. Treating children with early-onset conduct problems: a comparison of child and parent training interventions. *J Consult Clin Psychol*. 1997;65(1):93. doi:10.1037/0022-006X.65.1.93 9103739

[19] Holmes C, Levy M, Smith A, Pinne S, Neese P. A model for creating a supportive trauma-informed culture for children in preschool settings. *J Child Fam Stud*. 2015;24(6):1650–1659. doi:10.1007/s10826-014-9968-6 25972726 PMC4419190

[20] Vitaro F, De Civita M, Pagani L. The impact of research-based prevention programs on children’s disruptive behavior. *Exceptionality Educ Canada*. 1995;5:105–136.

[21] Webster-Stratton C. Early intervention for families of preschool children with conduct problems. The effectiveness of early intervention; 1997:429–453.

[22] Poulou MS. Emotional and behavioural difficulties in preschool. *J Child Fam Stud*. 2015;24(2):225–236. doi:10.1007/s10826-013-9828-9

[23] Mash EJ. *Treatment of Child and Family Disturbance: A Cognitive-Behavioral Systems Perspective*. The Guilford Press; 2006.

[24] Bruni O, Malorgio E, Doria M, et al. Changes in sleep patterns and disturbances in children and adolescents in Italy during the Covid-19 outbreak. *Sleep Med*. 2021;91:166–174. doi:10.1016/j.sleep.2021.02.003 33618965 PMC7871805

[25] Fergusson DM, Horwood LJ, Ridder EM. Show me the child at seven: the consequences of conduct problems in childhood for psychosocial functioning in adulthood. *J Child Psychol Psychiatry*. 2005;46(8):837–849. doi:10.1111/j.1469-7610.2004.00387.x 16033632

[26] Odgers CL, Caspi A, Broadbent JM, et al. Prediction of differential adult health burden by conduct problem subtypes in males. *Arch Gen Psychiatry*. 2007;64(4):476–484. doi:10.1001/archpsyc.64.4.476 17404124

[27] McGee R, Prior M, Willams S, Smart D, Sanson A. The long-term significance of teacher-rated hyperactivity and reading ability in childhood: findings from two longitudinal studies. *J Child Psychol Psychiatry*. 2002;43(8):1004–1017. doi:10.1111/1469-7610.00228 12455922

[28] Jakobsen IS, Fergusson D, Horwood JL. Early conduct problems, school achievement and later crime: findings from a 30-year longitudinal study. *New Zealand J Educ Stud*. 2012;47(1):123–135.

[29] Ramrakha S, Bell ML, Paul C, Dickson N, Moffitt TE, Caspi A. Childhood behavior problems linked to sexual risk taking in young adulthood: a birth cohort study. *J Am Acad Child Adolesc Psychiatry*. 2007;46(10):1272–1279. doi:10.1097/chi.0b013e3180f6340e 17885568

[30] Mathiesen KS, Sanson A. Dimensions of early childhood behavior problems: stability and predictors of change from 18 to 30 months. *J Abnorm Child Psychol*. 2000;28(1):15–31. doi:10.1023/a:1005165916906 10772347

[31] Moore KA, Evans VJ, Brooks-Gunn J, Roth J. What are good child outcomes. In: *The Well-Being of Children and Families: Research and Data Needs*. University of Michigan Press; 2001:59–84.

[32] Pace LE. Coaching parents to use positive behavior support: function-based interventions for preschool children with challenging behavior. Doctoral dissertation, Utah State University; 2019.

[33] Clark R, Menna R, Manel WS. Maternal scaffolding and children’s social skills: a comparison between aggressive preschoolers and non-aggressive preschoolers. *Early Child Dev Care*. 2013;183(5):707–725. doi:10.1080/03004430.2012.685935

[34] Hetherington EM, Bridges M, Insabella GM. What matters? What does not? Five perspectives on the association between marital transitions and children’s adjustment. *Am Psychol*. 1998;53(2):167. doi:10.1037/0003-066X.53.2.167 9491746

[35] Howlin P. Psychological and educational treatments for autism. *J Child Psychol Psychiatry Allied Discip*. 1998;39(3):307–322. doi:10.1017/S0021963097002138 9670087

[36] Fox L, Carta J, Strain PS, Dunlap G, Hemmeter ML. Response to intervention and the pyramid model. *Infants Young Child*. 2010;23(1):3–13. doi:10.1097/IYC.0b013e3181c816e2

[37] Steed EA, Shapland D. Adapting social emotional multi-tiered systems of supports for kindergarten classrooms. *Early Child Educ J*. 2020;48(2):135–146. doi:10.1007/s10643-019-00996-8

[38] Mendez M, Simpson T, Alter A, Meyers J. The infant mental health workforce: key to promoting the healthy social and emotional development of children. Impact: Ideas and Information to Promote the Health of Connecticut’s Children. Hartford: Connecticut Office of Early Childhood; 2015.

[39] Bryan J, Henry L. A model for building school–family–community partnerships: principles and process. *J Couns Dev*. 2012;90(4):408–420. doi:10.1002/j.1556-6676.2012.00052.x

[40] Hemmeter ML, Ostrosky MM, Corso RM. Preventing and addressing challenging behavior: common questions and practical strategies. *Young Except Child*. 2012;15(2):32–46. doi:10.1177/1096250611427350

[41] Hemmeter ML, Ostrosky M, Fox L. Social and emotional foundations for early learning: a conceptual model for intervention. *School Psych Rev*. 2006;35(4):583–601. doi:10.1080/02796015.2006.12087963

[42] Denham SA, Blair KA, DeMulder E, et al. Preschool emotional competence: pathway to social competence? *Child Dev*. 2003;74(1):238–256. doi:10.1111/1467-8624.00533 12625448

[43] Joseph GE, Strain PS. Comprehensive evidence-based social—emotional curricula for young children: an analysis of efficacious adoption potential. *Topics Early Child Spec Educ*. 2003;23(2):62–73. doi:10.1177/02711214030230020201

[44] Hieneman M, Dunlap G, Kincaid D. Positive support strategies for students with behavioral disorders in general education settings. *Psychol Sch*. 2005;42(8):779–794. doi:10.1002/pits.20112

[45] Hawken LS, Adolphson SL, Macleod KS, Schumann J. Secondary-tier interventions and supports. In: *Handbook of Positive Behavior Support*. Boston, MA: Springer; 2009:395–420.

[46] Denne LD, Gore NJ, Hughes JC, Toogood S, Jones E, Brown FJ. Implementing evidence-based practice: the challenge of delivering what works for people with learning disabilities at risk of behaviours that challenge. *Tizard Learn Disabil Rev*. 2020;25(3):133–143. doi:10.1108/TLDR-05-2020-0009

[47] Grey IM, McClean B, Barnes-Holmes D. Staff attributions about the causes of challenging behaviours: effects of longitudinal training in multi-element behaviour support. *J Learn Disabil*. 2002;6(3):297–312. doi:10.1177/1469004702006003037

[48] Tam S, McKay A, Sloan S, Ponsford J. The experience of challenging behaviours following severe TBI: a family perspective. *Brain Inj*. 2015;29(7–8):813–821. doi:10.3109/02699052.2015.1005134 25914927

[49] Whitlock EP, Orleans CT, Pender N, Allan J. Evaluating primary care behavioral counseling interventions: an evidence-based approach. *Am J Prev Med*. 2002;22(4):267–284. doi:10.1016/S0749-3797(02)00415-4 11988383

[50] Turnbull A, Edmonson H, Griggs P, et al. A blueprint for schoolwide positive behavior support: implementation of three components. *Except Child*. 2002;68(3):377–402. doi:10.1177/001440290206800306

[51] Bradshaw CP, Mitchell MM, Leaf PJ. Examining the effects of schoolwide positive behavioral interventions and supports on student outcomes: results from a randomized controlled effectiveness trial in elementary schools. *J Posit Behav Interv*. 2010;12(3):133–148. doi:10.1177/1098300709334798

[52] O’Neill RE, Albin RW, Storey K, Horner RH, Sprague JR. *Functional Assessment and Program Development*. Cengage Learning; 2014.

[53] O’Neill S, Stephenson J. The use of functional behavioural assessment for students with challenging behaviours: current patterns and experience of Australian practitioners. *Aust J Educ Dev Psyc*. 2010;10:65–82.

[54] Gresham FM, Watson TS, Skinner CH. Functional behavioral assessment: principles, procedures, and future directions. *School Psych Rev*. 2001;30(2):156–172. doi:10.1080/02796015.2001.12086106

[55] Bellone KM, Dufrene BA, Tingstrom DH, Olmi DJ, Barry C. Relative efficacy of behavioral interventions in preschool children attending head start. *J Behav Educ*. 2014;23(3):378–400. doi:10.1007/s10864-014-9196-6

[56] Blair KC, Fox L, Lentini R. Use of positive behavior support to address the challenging behavior of young children within a community early childhood program. *Topics Early Child Spec Educ*. 2010;30(2):68–79. doi:10.1177/0271121410372676

[57] Fettig A, Barton EE. Parent implementation of function-based intervention to reduce children’s challenging behavior: a literature review. *Topics Early Child Spec Educ*. 2014;34(1):49–61. doi:10.1177/0271121413513037

[58] Fettig A, Ostrosky MM. Functional assessment based parent intervention in reducing children’s challenging behaviors: exploratory study of group training. *Child Dev Res*. 2014;2014:1–11. doi:10.1155/2014/656327

[59] Hinton V, Buchanan AM. Positive behavior interventions and support in a physical activity summer camp. *Phys Edu*. 2015;72(4):660. doi:10.18666/tpe-2015-v72-i4-7141

[60] Moreno G. Addressing challenging behaviours in the general education setting: conducting a teacher-based Functional Behavioural Assessment (FBA). *Education*. 2011;39(4):363–371.

[61] Hieneman M. Positive behavior support for individuals with behavior challenges. *Behav Anal Pract*. 2015;8(1):101–108. doi:10.1007/s40617-015-0051-6 27703893 PMC5048254

[62] Zirkel PA. State special education laws for functional behavioral assessment and behavior intervention plans. *Behav Disord*. 2011;36(4):262–278. doi:10.1177/019874291103600405

[63] Sugai G, Lewis-Palmer T, Hagan-Burke S. Overview of the functional behavioral assessment process. *Exceptionality*. 2000;8(3):149–160. doi:10.1207/S15327035EX0803_2

[64] Simonsen B, Sugai G. School-wide positive behavior support: a systems-level application of behavioral principles. American Psychological Association; 2009.

[65] Tanguay P. *Nonverbal Learning Disabilities at School: Educating Students with NLD, Asperger Syndrome and Related Conditions*. Jessica Kingsley Publishers; 2001.

[66] Prinz RJ, Miller GE. Family-based treatment for childhood antisocial behavior: experimental influences on dropout and engagement. *J Consult Clin Psychol*. 1994;62(3):645. doi:10.1037/0022-006X.62.3.645 8063993

[67] Kaiser B, Rasminsky JS. Opening the culture door. *Young Child*. 2003;58(4):53–56.

[68] Dunst CJ, Hamby D, Trivette CM, Raab M, Bruder MB. Everyday family and community life and children’s naturally occurring learning opportunities. *J Early Interv*. 2000;23(3):151–164. doi:10.1177/10538151000230030501

[69] Buschbacher PW, Fox L. Understanding and intervening with the challenging behavior of young children with autism spectrum disorder. ASHA; 2003. 10.1044/0161-1461(2003/018) 27764323

[70] Kaiser AP, Hancock TB. Teaching parents new skills to support their young children’s development. *Infants Young Child*. 2003;16(1):9–21. doi:10.1097/00001163-200301000-00003

[71] Lucyshyn JM, Horner RH, Dunlap G, Albin RW, Ben KR. *Positive Behavior Support with Families*. Paul H Brookes Publishing; 2002.

[72] Breiner H, Ford M, Gadsden VL. federal policies and investments supporting parents and children in the United States. In: *Parenting Matters: Supporting Parents of Children Ages 0–8*. National Academies Press (US); 2016. 27997088

[73] Roggman LA, Cook GA, Peterson CA, Raikes HH. Who drops out of early head start home visiting programs? *Early Educ Dev*. 2008;19(4):574–599. doi:10.1080/10409280701681870

[74] El Nokali NE, Bachman HJ, Votruba-Drzal E. Parent involvement and children’s academic and social development in elementary school. *Child Dev*. 2010;81(3):988–1005. doi:10.1111/j.1467-8624.2010.01447.x 20573118 PMC2973328

[75] Vygotsky LS. Socio-cultural theory. *Mind Society*. 1978;6:52–58.

[76] Thompson RB, Foster BJ, Kapinos JR. Poverty, affluence and the Socratic method: parents’ questions versus statements within collaborative problem-solving. *Lang Commun*. 2016;47:23–29. doi:10.1016/j.langcom.2015.11.003

[77] Cakic L, Marjanovic-Umek L. Methods used by mothers to help children during solving cognitive problem tasks: comparison between mothers of securely and insecurely attached preschool children. *Stud Psychol*. 2015;57(1):21. doi:10.21909/sp.2015.01.671

[78] Lundy BL, Fyfe G. Preschoolers’ mind‐related comments during collaborative problem‐solving: parental contributions and developmental outcomes. *Soc Dev*. 2016;25(4):722–741. doi:10.1111/sode.12176

[79] Garner PW, Dunsmore JC, Southam‐Gerrow M. Mother–child conversations about emotions: linkages to child aggression and prosocial behavior. *Soc Dev*. 2008;17(2):259–277. doi:10.1111/j.1467-9507.2007.00424.x

[80] Trabelsi K, Ammar A, Masmoudi L, et al.. Globally altered sleep patterns and physical activity levels by confinement in 5056 individuals. ECLB COVID-19 international online survey; 2021. 10.5114/biolsport.2021.101605 PMC8670812 34937958

[81] Trabelsi K, Ammar A, Masmoudi L, Boukhris O, Chtourou H, Bouaziz B; ECLB-COVID19 Consortium. Sleep quality and physical activity as predictors of mental wellbeing variance in older adults during COVID-19 lockdown: ECLB COVID-19 international online survey. *Int J Environ Res Public Health*. 2021;18(8):4329. doi:10.3390/ijerph18084329 33921852 PMC8073845

[82] Varma A, Dergaa I, Mohammed AR, et al. Covid-19 and diabetes in primary care–How do hematological parameters present in this cohort? *Expert Rev Endocrinol Metab*. 2021;16(3):147–153. doi:10.1080/17446651.2021.1909472 33818239

[83] Dergaa I, Varma A, Tabben M, et al. Organising football matches with spectators during the COVID-19 pandemic: what can we learn from the Amir Cup Football Final of Qatar 2020? A call for action. *Biol Sport*. 2021;38(4):677–681. doi:10.5114/biolsport.2021.103568 34937978 PMC8670791

[84] Musa S, Dergaa I, Abdulmalik MA, Ammar A, Chamari K, Saad HB. BNT162b2 COVID-19 Vaccine hesitancy among parents of 4023 young adolescents (12–15 Years) in Qatar. *Vaccines*. 2021;9(9):981. doi:10.3390/vaccines9090981 34579218 PMC8473301

[85] Dergaa I, Abdelrahman H, Varma A, et al. COVID-19 vaccination, herd immunity and the transition toward normalcy: challenges with the upcoming sports event. *Ann Appl Sport Sci*. 2021;9. doi:10.29252/aassjournal.976

[86] Varma A, Dergaa I, Ashkanani M, Musa S, Zidan M. Analysis of Qatar’s successful public health policy in dealing with the Covid-19 pandemic. *Int J Med Rev Case Rep*. 2021;5(2):6–11.

[87] Musa S, Al Baker W, Al Muraikhi H, Nazareno D, Al Naama A, Dergaa I. Wellness program within primary health care: how to avoid “no show” to planned appointments?–a patient-centred care perspective. *J Phys Act Health*. 2021;5(1):76–86. doi:10.5334/paah.90

[88] Dergaa I, Abubaker M, Souissi A, et al. Age and clinical signs as predictors of COVID-19 symptoms and cycle threshold value. *Libyan J Med*. 2021. doi:10.1080/19932820.2021.2010337 PMC8667934 34895104

[89] Dergaa I, Saad HB, Souissi A, Musa S, Abdulmalik MA, Chamari K. Olympic Games in COVID-19 times: lessons learned with special focus on the upcoming FIFA World Cup Qatar 2022. *Br J Sports Med*. 2022;56(12):654–656. doi:10.1136/bjsports-2021-105276 35232751

[90] Dergaa I, Musa S, Romdhani M, et al. FIFA World Cup 2022: what can we learn from the inspiring Tokyo 2020 Olympic Games held in COVID-19 times? *Biol Sport*. 2022;39(4):1073–1080. 10.5114/biolsport.2022.113293 PMC9536391 36247947

[91] Bangkok UN. Empowering students with disabilities during the COVID-19 crisis; 2020. Available from: https://bangkok.unesco.org/content/empowering-students-disabilities-during-covid-19-crisis. Accessed June 16, 2022.

[92] Wang L, Zhang Y, Chen L, et al. Psychosocial and behavioral problems of children and adolescents in the early stage of reopening schools after the COVID-19 pandemic: a national cross-sectional study in China. *Transl Psychiatry*. 2021;11(1):342. doi:10.1038/s41398-021-01462-z 34083509 PMC8172553

[93] Ben salem A, Aldahnaim LA, Dergaa I. School nursing during COVID-19 pandemic: a brief report with a special focus on qatar’s experience. *OAJBS*. 2021;5(1). doi:10.38125/OAJBS.000345

[94] Lee J. Mental health effects of school closures during COVID-19. *Lancet Child Adolesc Health*. 2020;4(6):421. doi:10.1016/S2352-4642(20)30109-7 32302537 PMC7156240

[95] Jackson AM, Mullican LA, Tse ZT, et al. Unplanned closure of public schools in Michigan, 2015–2016: cross‐sectional study on rurality and digital data harvesting. *J School Health*. 2020;90(7):511–519. doi:10.1111/josh.12901 32383235

[96] Hawryluck L, Gold WL, Robinson S, Pogorski S, Galea S, Styra R. SARS control and psychological effects of quarantine, Toronto, Canada. *Emerg Infect Dis*. 2004;10(7):1206. doi:10.3201/eid1007.030703 15324539 PMC3323345

[97] Decosimo CA, Hanson J, Quinn M, Badu P, Smith EG. Playing to live: outcome evaluation of a community-based psychosocial expressive arts program for children during the Liberian Ebola epidemic. *Global Ment Health*. 2019;6. doi:10.1017/gmh.2019.1 PMC6521133 31143464

[98] Brazendale K, Beets MW, Weaver RG, et al. Understanding differences between summer vs. school obesogenic behaviors of children: the structured days hypothesis. *Int J Behav Nutr Phys Act*. 2017;14(1):1–14. doi:10.1186/s12966-017-0555-2 28747186 PMC5530518

[99] Löfstedt P, García-Moya I, Corell M, et al. School satisfaction and school pressure in the WHO European region and North America: an analysis of time trends (2002–2018) and patterns of co-occurrence in 32 countries. *J Adolesc Health*. 2020;66(6):S59–S69. doi:10.1016/j.jadohealth.2020.03.007 32446610

[100] Parikh R, Sapru M, Krishna M, Cuijpers P, Patel V, Michelson D. “It is like a mind attack”: stress and coping among urban school-going adolescents in India. *BMC Psychol*. 2019;7(1):1–9. doi:10.1186/s40359-019-0306-z 31138306 PMC6540371

[101] Brooks SK, Webster RK, Smith LE, et al. The psychological impact of quarantine and how to reduce it: rapid review of the evidence. *lancet*. 2020;395(10227):912–920. doi:10.1016/S0140-6736(20)30460-8 32112714 PMC7158942

